# Head Orientation Influences Saccade Directions during Free Viewing

**DOI:** 10.1523/ENEURO.0273-22.2022

**Published:** 2022-12-15

**Authors:** Stephanie M. Reeves, Emily A. Cooper, Raul Rodriguez, Jorge Otero-Millan

**Affiliations:** 1 Herbert Wertheim School of Optometry and Vision Science, University of California Berkeley, Berkeley, 94720, CA; 2 Helen Willis Neuroscience Institute, University of California Berkeley, Berkeley, 94720, CA; 3 Department of Neurology, Johns Hopkins University, Baltimore, 21231, MD

**Keywords:** direction bias, eye movements, head tilt, saccades, vestibular, virtual reality

## Abstract

When looking around a visual scene, humans make saccadic eye movements to fixate objects of interest. While the extraocular muscles can execute saccades in any direction, not all saccade directions are equally likely: saccades in horizontal and vertical directions are most prevalent. Here, we asked whether head orientation plays a role in determining saccade direction biases. Study participants (*n* = 14) viewed natural scenes and abstract fractals (radially symmetric patterns) through a virtual reality headset equipped with eye tracking. Participants’ heads were stabilized and tilted at −30°, 0°, or 30° while viewing the images, which could also be tilted by −30°, 0°, and 30° relative to the head. To determine whether the biases in saccade direction changed with head tilt, we calculated polar histograms of saccade directions and cross-correlated pairs of histograms to find the angular displacement resulting in the maximum correlation. During free viewing of fractals, saccade biases largely followed the orientation of the head with an average displacement value of 24° when comparing head upright to head tilt in world-referenced coordinates (*t*_(13)_ = 17.63, *p* < 0.001). There was a systematic offset of 2.6° in saccade directions, likely reflecting ocular counter roll (OCR; *t*_(13)_ = 3.13, *p* = 0.008). When participants viewed an Earth upright natural scene during head tilt, we found that the orientation of the head still influenced saccade directions (*t*_(13)_ = 3.7, *p* = 0.001). These results suggest that nonvisual information about head orientation, such as that acquired by vestibular sensors, likely plays a role in saccade generation.

## Significance Statement

We show that the statistics of saccade directions, from data collected during free viewing of fractal (radially symmetric) and natural scene images, are influenced by head orientation. During fractal viewing, saccade directions largely followed the orientation of the head with systematic offsets likely explained by ocular counter roll (OCR). During natural scene viewing of an Earth upright image, saccade directions were still influenced by head orientation. These results suggest that head and retinal orientation relative to gravity play a key role in saccade generation. Future work should consider the influence of head orientation when predicting saccade landing points or when using existing saccade generation models.

## Introduction

While saccades can be made in any direction, saccades in the cardinal directions are more prevalent than the oblique directions, and saccades in the horizontal direction are more prevalent than the vertical direction. This saccade direction bias is well documented and has been observed in tasks such as visual search (Gilchrist and Harvey, 2006; Najemnik and Geisler, 2008), movie watching (Costela and Woods, 2019), fixation (Otero-Millan et al., 2013), and free viewing (Foulsham et al., 2008; Tatler and Vincent, 2009; Otero-Millan et al., 2013; Anderson et al., 2020; Bischof et al., 2020).

There are many factors that likely contribute to the saccade direction bias, with oculomotor, neural, environmental, and behavioral origins. For example, purely horizontal saccades require the activation of fewer extraocular muscles and brain regions than saccades in other directions (Leigh and Zee, 2015); scenes have prevalent cardinal (especially horizontal) contour orientation biases that influence perception and saccade directions (Foulsham et al., 2008; Girshick et al., 2011; Raman and Sarkar, 2017; Rolfs and Schweitzer, 2022); and learned, directionally-biased behaviors such as reading may reinforce motor biases throughout the lifespan (Abed, 1991; Van Renswoude et al., 2016).

An additional contributor to the saccade direction bias that has not yet been systematically examined is the influence of gravitational signals indicating head orientation. Head tilt is a powerful tool to determine the relative contribution of gravitational signals because tilting the head disrupts the alignment between the direction of gravity and the head. When humans are upright, the visual world, gravity, head, and eyes are all in alignment, but during head tilt in the roll direction, the head is no longer aligned with the visual world or gravity. Moreover, ocular counter roll (OCR), occurring in response to head tilt, rotates the eye in the opposite direction of the head and breaks the alignment between the head and the retina.

There are many reasons why gravitational signals indicating head tilt may play a role in saccade generation. Previous studies have found that the perception of upright changes following head tilt (De Vrijer et al., 2009). This change in the perception of upright can be independent of changes in OCR (Otero-Millan and Kheradmand, 2016). The oblique effect, a perceptual effect characterized by enhanced discrimination for horizontal and vertical orientations, is also influenced by gravity and weakens when lying supine (Mikellidou et al., 2015). Other studies have used changes in head orientation to understand the underlying reference frame of perceptual and motor biases seen in tasks measuring visual acuity and stereoacuity (Ebenholtz and Walchli, 1965; Banks and Stolarz, 1975; Lam et al., 2008). Whether the effects of head orientation on vision can be completely explained by OCR is an active area of research (Banks and Stolarz, 1975; Medendorp et al., 2002; Klier et al., 2005). Given the influence of head orientation on visual perception and performance, it is likely that head orientation similarly influences saccade generation and saccade execution.

**Figure 1.**
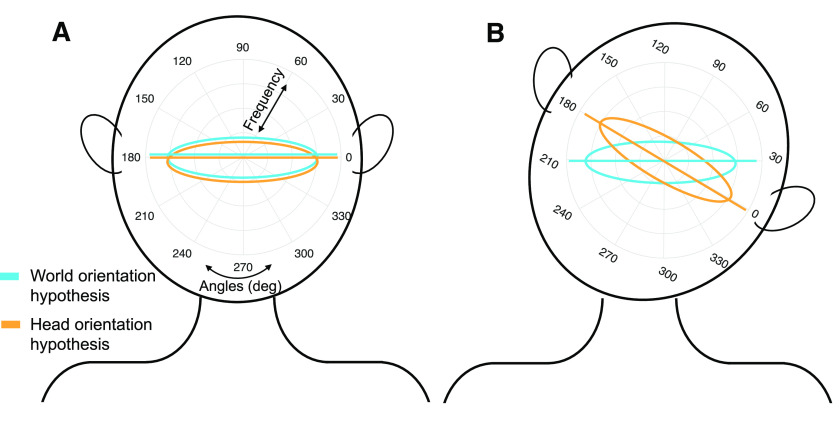
Predictions for the horizontal saccade direction bias as a function of head tilt. Polar histograms indicate the frequency of each saccade direction as a function of angle in degrees. ***A***, When the head is upright, the horizontal saccade bias looks the same for both the world orientation hypothesis and the head orientation hypothesis. ***B***, When the head is tilted, it is possible that the saccade bias will either rotate with the head (head orientation hypothesis) or stay consistent with the world and Earth upright (world orientation hypothesis). Blue and orange lines indicate reference lines (i.e., the horizontal axis) for the world and head orientation hypotheses, respectively.

In the present study, we examined whether roll tilting the head influences saccade direction distributions during free viewing. For clarity, we focus on the primary horizontal bias of saccade directions, but we expect the weaker vertical bias to also be present. Figure 1 shows the two alternative hypotheses for the effect of head tilt on saccade direction biases. First, according to the “world orientation hypothesis” (blue), saccade directions will remain primarily horizontal with respect to the world despite intervening head tilt. Second, according to the “head orientation hypothesis” (orange), the saccade bias will rotate with the head and remain horizontal with respect to the head. We first test the impact of head orientation on saccade directions when people visually explore images absent of visual cues of upright (fractal images), and ask which hypothesis is supported. Next, we examine the impact of head orientation during the viewing of Earth upright natural scenes. Finally, we show that our paradigm replicates previous work showing the effect of natural scene tilt by itself.

## Materials and Methods

### Participants

Fourteen adults (ages 22–38 with mean of 27 years; seven female, six male, and one nonbinary individual) from the community in and around Berkeley, CA participated in the study. All participants provided informed consent before data collection. The research followed the tenets of the Declaration of Helsinki, and the Institutional Review Board of the university approved the study.

Before conducting the study, we implemented an a priori one-tailed *t* test power analysis using G*Power (Faul et al., 2007), which revealed that at least 12 participants were required for the study based on an effect size of 0.8 (supported by pilot data that showed 7.7° of effect and 9.7° SD), an α of 0.05, and a power of 0.8.

### Apparatus

Stimuli were presented on a FOVE 0 Virtual Reality headset, controlled with a desktop computer. The FOVE (FOVE Inc) has a display resolution of 2560 × 1440 pixels, a field of view up to 100°, a weight of 520 g, and a frame rate of 70 Hz. The built-in binocular eye tracker uses a stereo infrared system that runs at 120 Hz. The FOVE measures head position and head rotation with an external infrared camera and a built-in inertial measurement unit (IMU), respectively.

The virtual space for stimulus presentation was created in Unity (version 2019.4.18f; Unity Technologies) and experimental structure was created with the Unity Experiment Framework, UXF version 2.1.1 (Brookes et al., 2020), which allows for the automation of data collection and data output. Eye movements were recorded using the Unity FOVE plugin (version 4.1.0). Custom scripts were written in C# to run the experiment.

**Figure 2.**
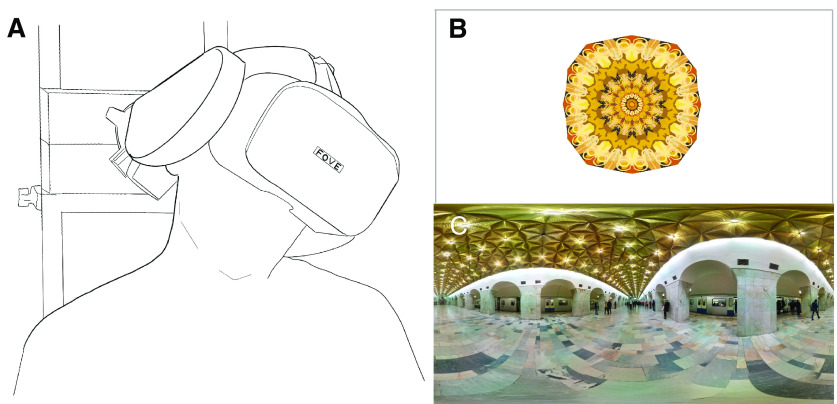
***A***, Head stabilization system for the FOVE virtual reality headset. ***B***, Example fractal photosphere image. ***C***, Example scene photosphere image licensed by Artem Svetlov and obtained from Flickr. The Flickr image was released with a CC-BY-2.0 license.

The head stabilizing system consisted of adjustable pads that gently held and compressed the temporal sides of the head (Fig. 2*A*). These pads were mounted to a rotating device that was able to roll tilt 360° and lock in place. Participants were held in place with the system and were released from the head stabilizers after every head tilt block (∼20 min).

### Stimuli

The stimuli consisted of 30 abstract fractals (Fig. 2*B*) and 30 natural scenes (indoor and outdoor scenes; Fig. 2*C*). The natural scenes were downloaded from Flickr and had CC-BY-2.0 licenses at minimum. The fractals were created with Procreate (Savage Interactive Ltd, Tasmania) on an iPad Pro (second generation, 11-inch). The fractals had 30° of radial symmetry and appeared in an amalgam of patterns and colors. Both scenes and fractal images were converted into equirectangular projection and then projected onto the inside of a sphere so that participants were completely immersed in the scene (i.e., the stimulus took up the entire field of view of the FOVE). The sphere had a diameter of 20 virtual meters. Although the FOVE allows for stereoscopic displays, the stimuli used in this study did not contain binocular or motion depth cues.

### Procedure

The experiment consisted of one session broken up into three blocks, one for each head tilt. At the beginning of each block and after eye movement calibration, the participant’s head was tilted and constrained at −30° (head tilt), 0° (head upright), or 30° (head right) rotation. Within each block, there were 60 free viewing trials that displayed a different image (30 natural scenes and 30 fractals) at −30°, 0°, or 30° relative to the head for a total of 180 trials in the session. Relative to the world, images could be tilted up to 60 or −60° for conditions where the image tilt and the head tilt were in the same direction. The image type and image tilt for each trial were presented in a random order within each head tilt block. Thus, within each head tilt block the images presented at different image tilts were all different. The head tilt block order was preset and balanced across participants.

At the start of the session, participants were prompted to complete a FOVE calibration consisting of a moving dot following an expanding spiral path. After successful calibration, the experimenter tilted the participant’s head the desired amount (−30°, 0°, or 30°, block dependent). Participant head tilt was monitored with an external digital angle gauge and an internal head rotation measurement exported by the FOVE while the experiment took place. Subjects maintained an average head tilt of 27.14 ± 3.66° (mean ± SD) to the right, 0.19 ± 2.24° upright, and −26.68 ± 2.37° to the left. The FOVE was configured so that the scene remained static in the head mounted display regardless of head movements.

Participants fixated a central dot and initiated each trial with a key press. After the button press, the fixation dot disappeared and was replaced with a natural scene or fractal. Participants had 15 s to explore the scene with their eyes without moving their head. After the allotted time, the image disappeared and was replaced with the central fixation dot. Every 20 trials, a white screen appeared, and participants initiated a calibration sequence with a space bar press. A blue dot appeared for 2 s at five different locations, and participants were instructed to follow the dot with their eyes.

### Data analyses and statistics

**Figure 3.**
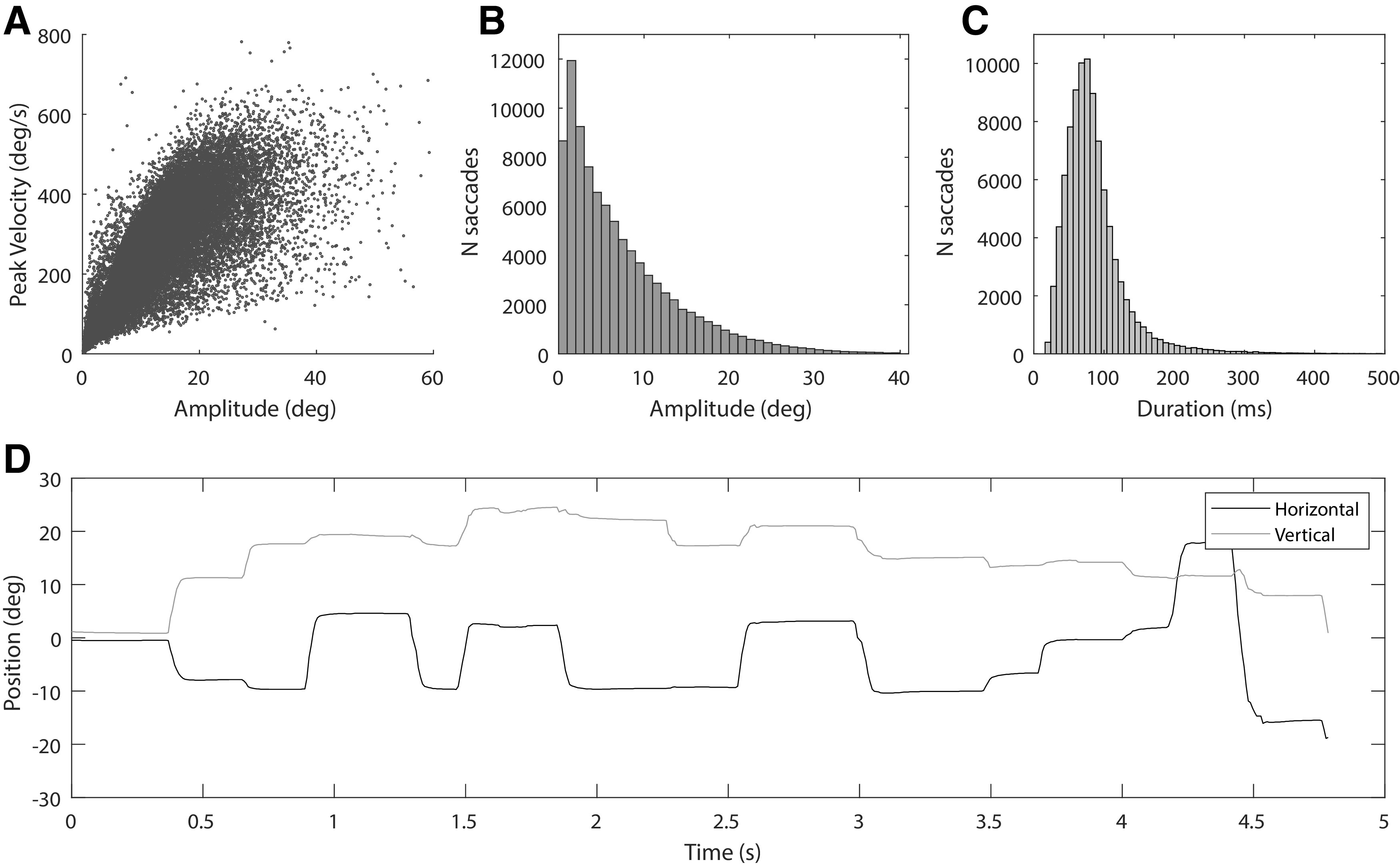
***A***, Main sequence of all saccades showing the stereotyped relationship between peak velocity and amplitude. ***B***, Histogram showing the number of saccades as a function of amplitude. ***C***, Histogram showing the number of saccades as a function of duration. ***D***, Horizonal and vertical eye traces for one subject during one trial.

Binocular eye movement data were exported from the FOVE and analyzed in MATLAB. Saccade detection was implemented using custom-built MATLAB functions that were based on the method described by Engbert and Kliegl (2003). Instantaneous velocity was calculated with a differential smoothing filter for each eye. Velocity thresholds for saccade detection were determined based on the robust standard deviation of the data using a λ of 8 (λ is a parameter in the Engbert algorithm that represents a multiplier of the standard deviation). We confirmed that detected saccades followed the main sequence (Fig. 3*A*) and other known characteristics such as the bias toward smaller (Fig. 3*B*) and shorter duration (Fig. 3*C*) saccades during free viewing. Eye movement traces were visually inspected for any abnormalities (Fig. 3*D*).

**Figure 4.**
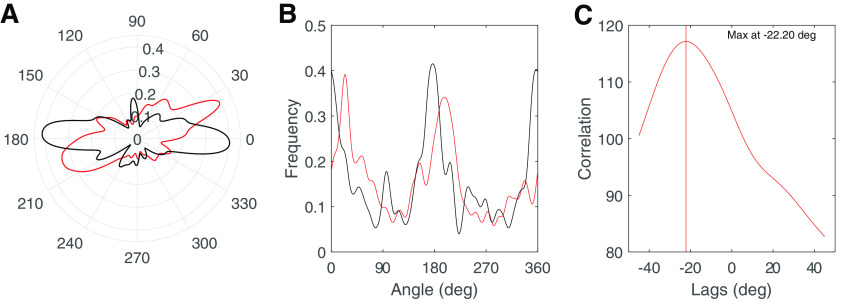
***A***, Polar distributions for a single subject during free viewing of fractal images for head tilt right (red) and head upright (black). The angles of the polar plots represent degrees while the radius indicates normalized frequency. ***B***, The unwrapped polar distributions from ***A***. ***C***, The output of the circular cross-correlation that identifies the lag (in degrees) that results in the maximum correlation.

We calculated polar distributions of saccade directions by applying a circular kernel density estimate (KDE) to the data (Muir, 2022). The kernel, a wrapped Gaussian 0.1 radians in width, was applied from 0° to 360° in steps of 0.1°. To quantify the differences among subsets of polar distributions, for each subject, we obtained circular cross-correlation values between pairs of polar distributions (see Results) and found the direction distribution displacement in degrees (Δ) that resulted in the maximum correlation (Fig. 4). To avoid edge effects, the circular cross-correlation, was implemented by repeating and concatenating the kernel density estimate three times so as to cover the range from −360° to 720° instead of only 0° to 360° and searching for a maximum correlation within a ±45° range to avoid finding spurious peaks given the 180° or 90° symmetry of the distributions. Implementing the circular cross-correlation was made possible by the fact that individual subjects had saccade direction distributions that were anisotropic; thus, the lag that resulted in the maximum correlation was indeed the highest (Fig. 4). These direction distribution displacements for individual subjects were bootstrapped to determine 95% confidence intervals (CIs). A positive direction distribution displacement value indicates a clockwise rotation of the saccade bias while a negative value indicates a counterclockwise rotation of the bias.

The reference frame index (RFindex) was calculated for each subject according to the equation $RFindex=\frac{\Delta_{30}+(-\Delta_{-30})/2}{T}$ where Δ represents the direction distribution displacement between 30° and 0° tilt ( Δ30) and between −30° and 0° tilt ( Δ−30), and T indicates the absolute average tilt of either the head or image tilt in degrees associated with a given condition. That is, for each subject, we sign-reversed the direction distribution displacement values corresponding to head or image tilt towards the left, calculated the mean direction distribution displacement for left and right tilt, and then scaled the values by the absolute average tilt associated with the experimental condition. A RFindex of 0 corresponds with a saccade bias that is oriented to a head reference frame while a RFindex of 1 corresponds with a world or gravity fixed reference frame (see Results for exceptions). The RFindex allows us to combine the data from left and right tilts and summarize it into a single number to test the overall significance of our effects.

We were able to obtain the torsional component of recorded eye movements for 11 of the 14 subjects. We analyzed OCR during our study by calculating the median OCR over time for each trial to reduce the effect of outliers, and then calculating the average OCR for each subject, head tilt, and image type. We calculated the change in OCR between head tilts for fractal viewing by subtracting the OCR during head tilt right from the OCR during head upright and by subtracting the OCR during head tilt left from the OCR during head upright.

## Results

### Effect of head tilt on saccade direction distributions while free viewing fractal images

Our main research question was whether saccade direction distributions would rotate as a function of head tilt (head orientation hypothesis) or stay consistent with the environmental upright (world orientation hypothesis; Fig. 1). For this question, we examined eye movements during trials showing radially symmetric fractal images since these fractals do not contain directional cues or other semantic content.

**Figure 5.**
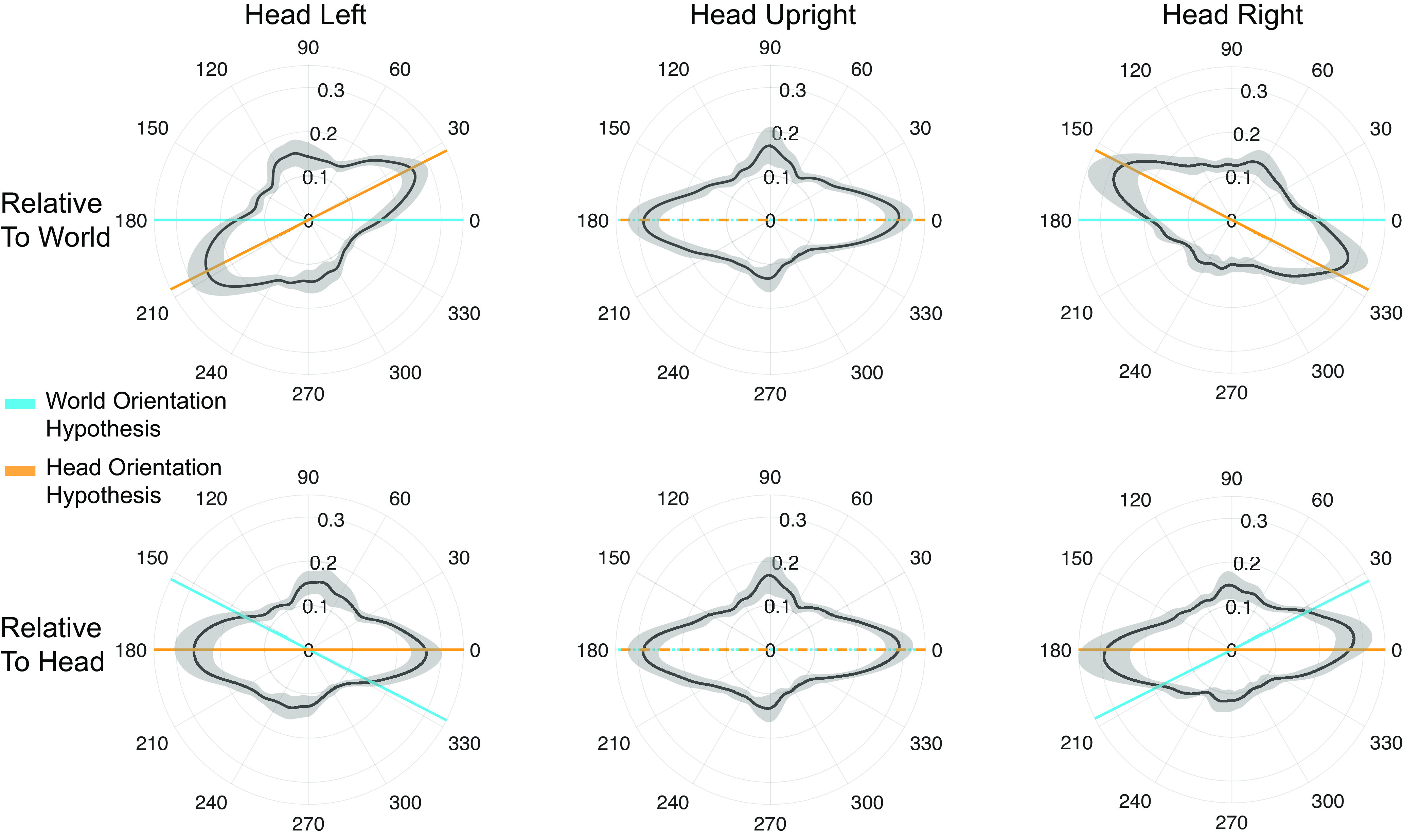
Saccade direction distributions during free viewing of fractal images for head tilt left, head upright, and head tilt right. The top row shows the data relative to the world (i.e., accounting for subject head tilt), while the bottom row shows the data relative to the head. Each polar plot is calculated by applying a circular kernel density estimate on saccade direction data. The angles of the polar plots represent degrees while the radius indicates normalized frequency. The black histogram line indicates the mean across subjects while the shaded gray indicates 95% CIs. The blue and orange lines indicate the orientation of the world and head hypotheses, respectively, showing the average measured head tilt.

Figure 5 displays the three polar distributions (one for each head tilt) in both world-referenced coordinates (top row) and head-referenced coordinates (bottom row). The saccade distributions plotted in Figure 5 clearly rotate with the orientation of the head and are not world-fixed. In world coordinates (top row), the average direction distribution displacement (obtained from the cross-correlation technique described in Materials and Methods) across all subjects was 24.13° (95% CI [20.75, 27.51]) between head upright and head right, which was significantly different from the average −24.22° (95% CI [−27.87, −20.56]) displacement between head upright and head left (*t*_(13)_ = −21.13, *p* < 0.001; Cohen’s *d* = 5.65). This result indicates that saccade directions during free viewing of fractal images are influenced by head orientation and do not follow the world orientation hypothesis.

**Figure 6.**
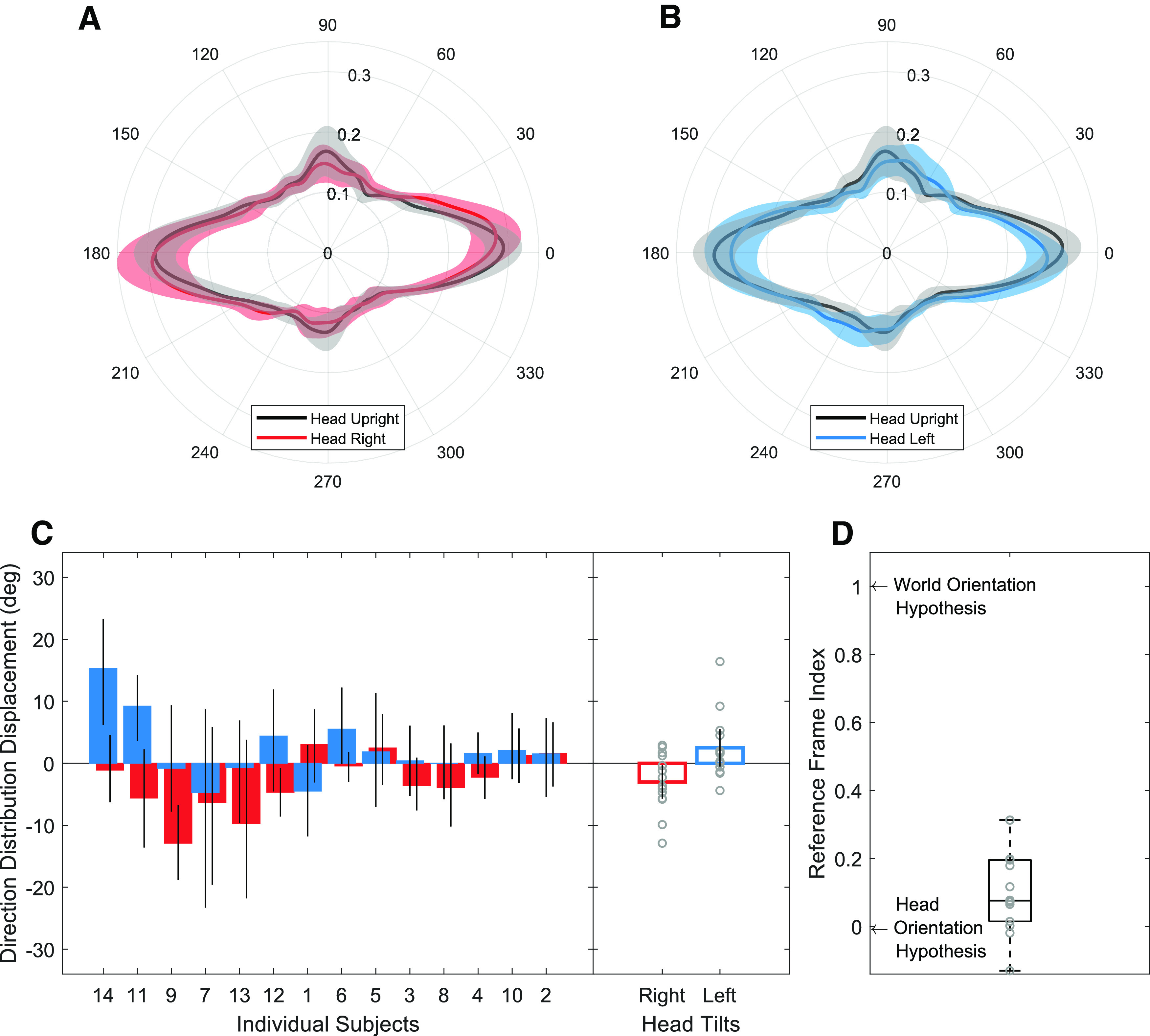
Saccade polar histograms for all subjects during fractal viewing, shown in head coordinates, for (***A***) head upright and head tilt right distributions and (***B***) head upright and head tilt left distributions. ***C***, Left, Bootstrapped average displacements for individual subjects with 95% CI error bars. Right, Bar plots showing the direction distribution displacement for all subjects. Circles represent individual data and error bar represents 95% CIs. ***D***, Box and whisker plot showing the reference frame index derived by scaling the direction distribution displacement values for each subject by their head tilt amount in degrees.

After rejecting the world-orientation hypothesis, we next asked to what extent saccade directions follow the head orientation hypothesis. In head coordinates (Figs. 5, 6*A*,*B*, bottom row), the average angular direction distribution displacement across all subjects was −3.02° (95% CI [−5.70, −0.33]) between head upright and head right and 2.47° (95% CI [−0.52, 5.48]) between head upright and head left. The average RFindex for these data were 0.10 (Fig. 6*D*), which was significantly different from zero (*t*_(13)_ = 3.12, *p* = 0.008, Cohen’s *d* = 0.84) and confirms that the head orientation hypothesis can be rejected. This indicates that saccade directions do not perfectly follow the head orientation and that there is some degree of offset that may originate from ocular counter roll.

**Figure 7.**
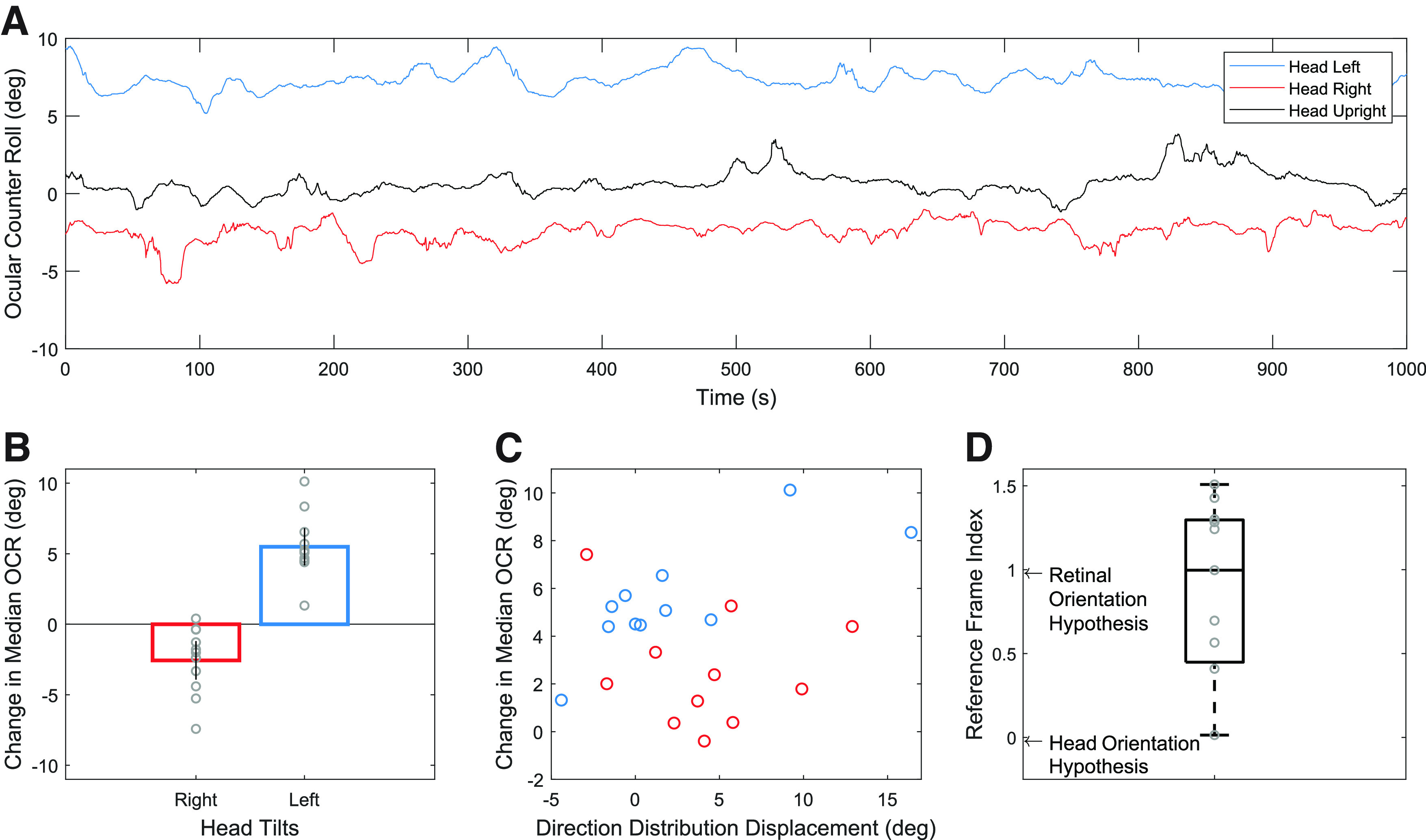
***A***, Ocular counter roll traces from one subject from head tilt left, head upright, and head tilt right conditions. ***B***, Change in median OCR values for each subject derived by subtracting the median OCR value of head tilt left (or right) from the median OCR value of head upright. Circles represent individual subjects. Error bars indicate 95% CIs. ***C***, Change in median OCR as a function of the cross-correlation displacement values for each subject (negative values were reversed). ***D***, Reference frame index found by scaling the direction distribution displacement values for each subject by their change in median OCR amount in degrees.

These results suggest an alternative to the world-orientation or head-orientation hypothesis. During head tilt, ocular counter roll (OCR) brings the eye in the opposite direction of the head toward Earth upright. OCR only partially compensates for head tilt in humans, and asymptotes at around 8°−10°. It is thus possible that the saccade bias is generated with respect to a retinal or eye reference frame instead of a head reference frame. Indeed, the deviations observed relative to the head-orientation hypothesis for both tilt directions are qualitatively consistent with this hypothesis, so we explored it further. We aimed to analyze the eye tracking torsion data for all subjects to determine whether a retinal reference frame is the best explanation for the saccadic eye movements during free viewing of fractals. Figure 7*A* shows OCR traces for one subject while Figure 7*B* shows the change in median OCR values for all subjects where torsion was successfully measured (11 of 14 subjects). On average, the change in median OCR between head tilt right and upright was −2.57° (95% CI [−3.9, −1.2]) while the change in median OCR between head tilt left and upright was 5.49° (95% CI [4.2, 6.8]; Fig. 7*B*). The direction distribution displacement values, obtained with the cross-correlation technique described in Materials and Methods, and the median OCR values are roughly on the same order of magnitude (Fig. 7*C*). Although there is not a direct correlation (ρ = 0.21, *p* = 0.36), the study was not designed to have sufficient power to detect a correlation. To determine whether saccades are generated with a retinal reference frame, we calculated an additional reference frame index by scaling direction distribution displacement values by the change in median OCR. The average reference frame index for these data were 0.78 (Fig. 7*D*), which was significantly different from zero (indicating a head reference frame; *t*_(13)_ = 3.63, *p* = 0.005, Cohen’s *d* = 1.09) and not significantly different from one (indicating a retinal reference frame; *t*_(10)_ = 1.00, *p* = 0.34). This suggests that the orientation of the saccade direction bias during fractal viewing as a result of head tilt may be explained by OCR.

### Effect of head tilt on saccade direction distributions while free viewing a scene

**Figure 8.**
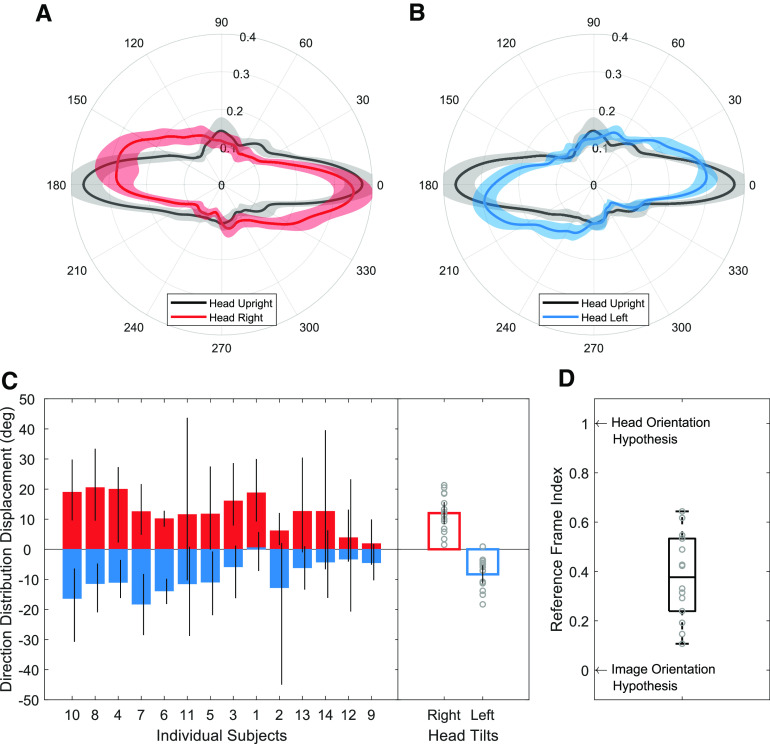
Saccade polar histograms for all subjects during Earth upright scene viewing, shown in world coordinates, for (***A***) head upright and head tilt right distributions and (***B***) head upright and head tilt left distributions. ***C***, Left, Bootstrapped average displacements for individual subjects with 95% CI bars. Right, Bar plots showing the direction distribution displacement for all subjects. Circles represent individual data and error bar represents 95% CIs. ***D***, Box and whisker plot showing the reference frame index derived by scaling the direction distribution displacement values for each subject by their head tilt amount in degrees.

We next asked whether saccade directions are influenced by head tilt when viewing an Earth upright scene. If the content of the scene is strong enough to change the orientation of the horizontal bias in response to scene tilt (Foulsham et al., 2008; Bischof et al., 2020), then one possibility is that the saccade distributions for all Earth upright scenes will look the same regardless of head orientation (we call this the “image orientation hypothesis”). However, if head or retinal orientation play a key role in saccade generation, then we would expect different distributions among head tilts. For this analysis, we calculated polar histograms in world coordinates (i.e., accounting for head tilt; Fig. 8*A*,*B*) and then implemented the cross-correlation procedure (see Materials and Methods) for each subject to obtain direction distribution displacements. We used the subset of conditions with Earth upright scenes for this analysis: namely, 30° natural scenes during head tilt left, 0° natural scenes during head upright, and −30° natural scenes during head tilt right. We found an average direction distribution displacement of 12.01° (95% CI [8.36, 15.66]) when comparing head tilt right and upright and −8.32° (95% CI [−11.35, −5.30]) when comparing head tilt left and upright (Fig. 8*C*). The average reference frame index was 0.38, which was significantly different from zero (indicating an image reference frame; *t*_(13)_ = 8.16, *p* < 0.001, Cohen’s *d* = 2.18) and one (indicating a head reference frame; *t*_(13)_ = 13.38, *p* < 0.001, Cohen’s *d* = 3.58). This result indicates that when individuals view an upright natural scene while their head is tilted, saccades do not precisely follow the orientation of the image or the orientation of the head. Instead, saccade directions fall somewhere in the middle, which suggests that head orientation matters even when viewing an Earth upright scene.

### Effect of scene tilt on saccade direction distributions

**Figure 9.**
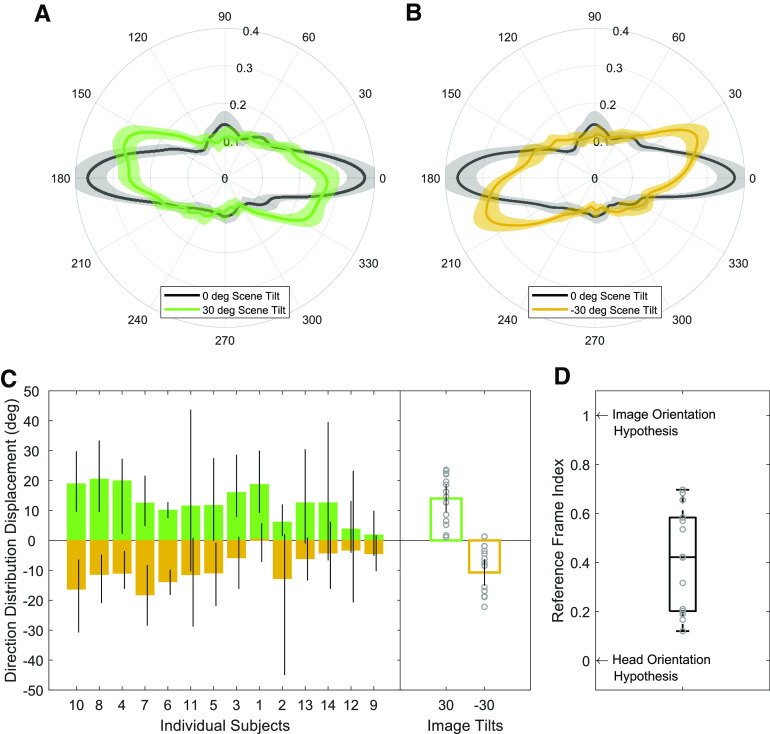
Saccade direction polar histograms during head upright for scenes with (***A***) 0° and 30° tilt and (***B***) 0° and −30° tilt. ***C***, Left, Bootstrapped average displacements for individual subjects with 95% CI bars. Right, Bar plots showing the direction distribution displacement for all subjects. Circles represent individual data and error bar represents 95% CIs. ***D***, Box and whisker plot showing the reference frame index derived by scaling the direction distribution displacement values for each subject by the scene tilt amount in degrees.

Previous work has shown that saccade directions follow the orientation of a natural scene. To confirm the influence of natural scene tilt on saccade directions while the head is upright, we calculated polar histograms (Fig. 9*A*,*B*) and measured the direction distribution displacements (Fig. 9*C*) between −30° and 0° scene tilts as well as 30° and 0° scene tilts. In our sample, we found that some subjects were very likely to make saccades following the orientation of the image while others were less likely. There was a mean direction distribution displacement of 14.02° (95% CI [9.33, 18.70]) when comparing 0° and 30° scene tilts and −10.71° (95% CI [15.09, −6.33]) when comparing 0° and −30° scene tilts (Fig. 9*C*). The reference frame index was 0.41 (Fig. 9*D*), which was significantly different from zero (indicating a head reference frame; *t*_(13)_ = 7.35, *p* < 0.001, Cohen’s *d* = 1.97) and significantly different from one (indicating an image reference frame; *t*_(13)_ = 10.48, *p* < 0.001, Cohen’s *d* = 2.80). This result suggests that saccade directions were made neither in direct alignment with the scene (that was Earth upright) nor the head (that was tilted). The exact reference frame here is somewhat ambiguous, perhaps in part because of the high variability across subjects in our data (Fig. 9*C*). We also compared the effect of image tilt relative to the head for all three head tilts and observed a comparable effect (*F*_(2,39)_ = 2.96, *p* = 0.06).

## Discussion

When humans free view scenes, saccades are more likely to occur in the cardinal directions and especially in the horizontal direction. It is unclear what happens to saccade direction biases in response to head tilt. We asked whether saccade biases remain fixed to a world reference frame, a head reference frame, or some other reference frame. To answer this research question, we used the saccade direction anisotropy to study the impact of head orientation on saccade generation by recruiting individuals to view fractal and natural scene images at three head orientations and measuring changes in the distribution of saccade directions.

When participants viewed fractals that contained radially-symmetric orientation cues, we found that saccade direction distributions remained largely fixed relative to head orientation. However, we found that saccade distributions were systematically offset from exact alignment with the head. This slight offset was of the same order of magnitude and direction as predicted by ocular counter roll (OCR). These results suggest that when strong orientation information is unavailable (such as when free viewing fractal images), saccades are generated with respect to an egocentric reference frame that is likely in retinal coordinates. Additionally, when participants viewed Earth upright natural scenes while tilting the head, we found that head orientation had a significant effect on saccade directions with saccade distributions falling in between a head reference frame and a world reference frame. Given previous work showing that the orientation of a scene influences the way we look at it (Foulsham et al., 2008; Anderson et al., 2020; Bischof et al., 2020), this finding clarifies that it is not just the orientation of the scene that matters, but also the orientation of the head and retina with respect to the gravity-driven world. From this analysis, we conclude that head orientation has an impact on saccade directions even when viewing an Earth upright scene.

As a confirmation of previous work, we also set out to examine the effect of scene tilt on saccade directions when the head is upright. In general, we found agreement with previous literature showing that saccade directions follow the orientation of the scene (Foulsham et al., 2008; Anderson et al., 2020; Bischof et al., 2020). However, while previous work appeared to show almost perfect alignment between saccade directions and scene orientation as shown qualitatively by polar histograms, our data revealed high subject variability such that some subjects made saccades that were closely aligned with the scene while others made saccades that were not aligned with the scene. It is currently unclear why this discrepancy occurs. One difference among studies is the angles of image tilt tested. While we only focused on a 30° tilt, previous studies used 45° and 90° tilts.

There are known biases in visual perception and scene statistics that might be related to the biases observed in saccade direction. In perception, the oblique effect corresponds with enhanced performance of the discrimination of horizontally and vertically oriented stimuli (Appelle, 1972; B Li et al., 2003), while the visual field effect shows biases in performance for stimuli located along the vertical and horizontal meridians (Carrasco et al., 2001). It could be that these perceptual biases are related to saccade motor biases in one of two ways: (1) the visual system could compensate for worse perception in the oblique directions, such as in the visual field effect, by generating more saccades in those directions, or (2) enhanced perception in horizontal and vertical directions may increase the likelihood of selecting a target and prompting a saccade in those directions. There is evidence for this second possibility in studies using gaze-contingent displays that shows that humans make more saccades in the direction where their vision is best (Foulsham et al., 2011). Researchers have hypothesized that these perceptual biases result from an optimal adaptation to scene statistics (Girshick et al., 2011). Indeed, orientation contours in natural scenes are strongest for the horizontal and vertical directions (Switkes et al., 1978). The saccade biases may be related directly to the statistics of natural scenes by increasing the power of parallel orientations in scenes during saccades (Rolfs and Schweitzer, 2022). Others have found that natural scene statistics and neural representations of natural images are critical factors in guiding saccades and saccade amplitudes across species (Samonds et al., 2018). Our analysis confirms previous work showing that scene orientation influences saccade directions, although it is unlikely that scene statistics are entirely responsible for the bias in saccade directions since the saccade bias is present even in the absence of a scene (Otero-Millan et al., 2013).

Previous studies have looked at the effect of head tilt on some of these perceptual biases and on how the brain encodes visual stimuli during head tilt. For example, Mikellidou (2015) examined whether the oblique effect is anchored to an allocentric or egocentric reference frame and found that the effect was allocentric when sitting upright (regardless of head tilt) but egocentric when lying supine. Banks and Stolarz (1975) also examined whether visual sensitivity (acuity) was grounded in allocentric or egocentric coordinates and found that the decrease in visual performance with head tilt was likely explained by OCR, which indicates that meridional visual acuity differences correspond to the retinal and not spatial orientation of the stimulus. Clearly the brain has access to multiple frames of reference, but it is unclear how this information is combined and used. While it is possible that saccades are generated retinotopically since cortical areas of the brain are retinotopically organized and receive retinotopic visual information, it is also possible that neurons store sensory and motor events in multiple reference frames simultaneously. For example, even when accounting for ocular torsion, neuronal receptive fields shift in early visual areas (Pouget et al., 2002; Daddaoua et al., 2014; Khazali et al., 2020). In this view, there is not a single reference frame that is or is not transformed, but rather multiple flexible reference frames that can be accessed in a variety of ways.

Determining the reference frame of saccade planning and execution has been a major subject of research for decades, and yet, to this day, we are still puzzled by the ways in which the brain encodes complex visual information and interprets it into a well-coordinated motor command. From animal, human, and computational studies, five general brain regions have been identified as contributing in a significant way to saccade generation: brain stem reticular formation saccadic burst generators, superior colliculus, cerebellum, basal ganglia, and premotor cortical areas (Girard and Berthoz, 2005). The overall task of all these brain areas is to select what saccade must be executed by integrating information from visual, vestibular, auditory, and proprioceptive sensory modalities that are encoded in different frames of reference. Whichever brain area or areas are the source of the biases in saccade generation must encode saccades with a mixture of egocentric (eye or head) and allocentric reference frames. Indeed, most studies of goal-related activity in the superior colliculus (SC), frontal cortex, and posterior parietal cortex (PPC) have shown that a gaze-centered retinal frame of reference is likely (Klier et al., 2001; Andersen and Buneo, 2002; Crawford et al., 2011), in alignment with the work presented here. However, allocentric maps must also be created and used in saccade generation since we know that saccade directions are influenced by the orientation of natural scenes and allocentric cues are important for gaze precision (J Li et al., 2017). Such allocentric maps could influence saccade direction biases through the alignment of salient or semantically important objects, through low-level visual statistics such as orientation energy, or even from high-level perception of gravitational upright. Recently, research has suggested that allocentric and egocentric maps are combined in the frontal eye fields (FEFs; Bharmauria et al., 2020), although other brain areas are likely involved as well.

In conclusion, we show that signals indicating head orientation can influence known statistics of saccade directions. These results suggest that head and retinal orientation relative to gravity play a key role in saccade generation. Head tilt is a valid tool that can be used to disambiguate the reference frame of saccade generation mechanisms by comparing retinal, head, and world coordinates. Moving forward, integrating head orientation information into models of saccade generation and target selection may improve our ability to understand and predict saccade patterns under natural viewing conditions.

## Synthesis

Reviewing Editor: David Schoppik, New York University Decisions are customarily a result of the Reviewing Editor and the peer reviewers coming together and discussing their recommendations until a consensus is reached. When revisions are invited, a fact-based synthesis statement explaining their decision and outlining what is needed to prepare a revision will be listed below. The following reviewer(s) agreed to reveal their identity: Tom Foulsham.

First off, apologies for the slow response; I’ve been traveling. We all agreed that the manuscript was a sensible set of experiments that were well-presented. The reviewers had a number of points, listed below, that we felt were worth addressing in a revision. Since everyone agreed that these were relatively minor points, I recommend attempting the reanalyses where practical and addressing the points in the text where appropriate.

1. The scene tilt conditions are not completely clear. There were 60 trials per head tilt block (I think), 30 per image type (line 135). These were also rotated, but were they rotated relative to the world/gravity or relative to the head? So a 30 tilted image in a 30 tilted block was actually at 60 degrees from it’s original orientation? In section 5.2 it suggests that scenes were “Earth upright", so is that only a subset of the conditions? Was each particular image repeated in these conditions for a particular participant? Although the predictions in Figure 1 etc are very nice, it seems like they have a third hypothesis where the saccade direction bias goes with the Scene orientation, so maybe this should be depicted as well?

2. It wasn’t always clear what was being analyzed and why the authors chose a given statistical approach given the underlying assumptions. I think that the t-tests on line 217 and elsewhere are performed on the results of a cross correlation. It would be better if the authors reminded about this in the results because it often sounds like the distributions are being compared directly. Could the authors reassure us that this technique captures the pattern they see in particular participants. How does their cross-correlation handle the fact that these are polar histograms (i.e., how do they avoid edge effects?). Some previous papers use a Von Mises distribution to capture the circular nature of these.

2a. One suggestion: Instead of binning saccade directions into bins of 10deg and then interpolating, it might be more precise to apply a kernel density estimate to the saccade directions (of course, this requires a KDE on circular data). Especially the head-orientation vs retina-orientation analysis is on a range where the bins are quite large compared to the effect size. But this point is only a suggestion, not a request.

2b. At the heart of all analyses in this paper is the distribution displacement, which is computed by maximizing the cross correlation between one distribution and another shifted distribution. Since this value is quite important, I think it should be be inspected a bit more to justify its informativeness. Two things come to mind first:

2a1. checking that the maximum correlation is actually high and that it’s higher than for other shifts. The plotted histograms suggest that that’s the case (which is why I’m not too worried about it), but maybe it should be discussed in the text that this could be a problem, but that it is (likely) not the case here.

2a2. Secondly, right now there is no error computed for the displacement, but in theory, there could be quite some error (especially if the distributions are close to uniform -- which they are not). The most natural way to compute an error range for the displacement that comes to my mind (but maybe the authors have an even better idea!) is as follows: We compute the binned histograms for both datasets as done before (let’s say for upright and +30deg). But now, before computing the displacement, we sample from both histograms new datapoints: exactly as many samples as the original data had, which went into said histogram. Then we can compute new histograms from the sampled data and again get two histograms, one for upright and one for +30deg. And now we compute the displacement value for these histograms from the sampled data. We can repeat the procedure of sampling, binning and computing of the displacement value many times and that way bootstrap an error interval for the displacement value. This error will tell us, how much we can rely on the displacement value given that we used only finite data to estimate the underlying distributions. Optimally, these error values would even propagated into the t-test, but that would require a different test and to be honest I’m not sure what the correct test would be, it might require doing Bayesian inference on a graphical model. So I guess it’s fine to keep the t-test as long as there is an discussion why we can trust the displacement values, for example via the mentioned computation of error ranges.

3. When the t-test comparisons are performed, it wasn’t clear why the comparison was upright and right vs. upright and left. Perhaps the effect of interest is just whether the displacement compared to upright is significantly different to zero, not whether this is different in left or right tilts.

4. In section 5.3, the RF index result is “somewhat ambiguous” in that it is half way between the image and head hypotheses. This is supported by two t-tests comparing it to 0 and 1 (both significant). In the previous section, only one of these tests is presented and the conclusion is more definitive - that’s a bit inconsistent. In the discussion, the authors suggest that their results are different from previous studies that showed a strong scene-centred bias. That is difficult to judge - have previous studies used the same statistical methods or RF index? What did they find? Is it the case that saccades don’t rotate as much as in previous studies, or that some people do and some people do not? I think it would be beneficial to plot all the conditions for the scene condition (I think that is a 3 x 3 design) so that readers can judge the effects of the two tilts.

5. Was there a reason this study was performed in VR? Would the same results be expected if images were just presented on a flat screen?

6. The significance statement seems more like a short summary to me. Not sure about expectation due to journal, but I would hope for something that reports not only the results, but why they are important/why I should read the paper. Only last sentence of the significance statement touches on this. But I might simply be misunderstanding what the significance statement is about.

7. I am not sure I correctly understood what Delta_+-30 is in the computation of the RFindex. From the previous section, I guess that Delta_alpha is the Delta between head-upright and head-tilt of alpha, i.e., the displacement in degree that you need to apply to the head-upright data to yield maximum crosscorrelation with the data from the head tilt of alpha? It might be helpful to introduce not only Delta, but also the subscripted version.

8. Right now, the results and the discussion mainly focuses on whether or not the tested effects are significant. I personally think that such a discussion should always be combined with a discussion of how large the effect is. For example, the authors correctly point out that they found an effect of head orientation on saccadic directions even in the scene viewing case (line 281 and line 328). But, unlike in the fractal viewing case, here the effect seems to be very small compared to the effect of the scene directions. This doesn’t make the results wrong or less interesting but I think it helps putting them into a bigger picture.

9. line 396, “However, allocentric maps must also be created and used in saccade generation since we know that saccade directions are influenced by the orientation of scenes and allocentric and egocentric maps are combined in the frontal eye field”. It seems the influence of scene orientation on saccadic directions could in theory also come purely from the alignment of interesting (“salient”) object in the scene. For example, if most interesting stuff is aranged roughly in a line, then most saccades have to be in the orientation of the line, independent on whether the saccades are created in allocentric or egocentric maps.

## Author Response

Computational Neuroscience Model Code Accessibility Comments for Author (Required):

The code should be accessible; please consider a repository or github where appropriate.

We have created a GitHub repository for our saccade data and scripts that conduct the analysis and figures/statistics code.

Synthesis Statement for Author (Required):

First off, apologies for the slow response; I’ve been traveling. We all agreed that the manuscript was a sensible set of experiments that were wel¬¬l-presented. The reviewers had a number of points, listed below, that we felt were worth addressing in a revision. Since everyone agreed that these were relatively minor points, I recommend attempting the reanalyses where practical and addressing the points in the text where appropriate.

Thank you to the editor and reviewers for their helpful comments on our manuscript. We hope that we have improved the manuscript by incorporating your suggestions and we look forward to the possibility of publication in eNeuro.

1. The scene tilt conditions are not completely clear. There were 60 trials per head tilt block (I think), 30 per image type (line 135). These were also rotated, but were they rotated relative to the world/gravity or relative to the head? So a 30 tilted image in a 30 tilted block was actually at 60 degrees from it’s original orientation? In section 5.2 it suggests that scenes were “Earth upright", so is that only a subset of the conditions? Was each particular image repeated in these conditions for a particular participant? Although the predictions in Figure 1 etc are very nice, it seems like they have a third hypothesis where the saccade direction bias goes with the Scene orientation, so maybe this should be depicted as well?

We apologize that the image tilt conditions were not clear. All image tilts were made relative to the head, therefore, a 30{degree sign} tilted image during a 30{degree sign} tilted block (head right) would indeed equate to a 60{degree sign} image tilt relative to Earth upright. In section 5.2, the Earth upright images that we refer to are a subset of conditions in which the combined head and image tilt result in an Earth upright image orientation, namely: 0{degree sign} natural scenes during head upright, 30{degree sign} natural scenes during head tilt left (-30{degree sign}), and -30{degree sign} natural scenes during head tilt right (30{degree sign}). Because we randomized 30 different natural images within the 30 trials in each head tilt block, the same image may or may not have been repeated in the three head tilt conditions with Earth upright orientation. We have updated the Methods (4.4, lines 144-154) and Results (5.2, lines 323-325) in the manuscript to make this information clearer.

We have also updated the Introduction to clarify the context of Figure 1, noting that the saccade direction bias as a function of head tilt is our main research question and that secondary analyses in the manuscript confirm previous results that saccade directions are influenced by the orientation of a natural scene.

2. It wasn’t always clear what was being analyzed and why the authors chose a given statistical approach given the underlying assumptions. I think that the t-tests on line 217 and elsewhere are performed on the results of a cross correlation. It would be better if the authors reminded about this in the results because it often sounds like the distributions are being compared directly. Could the authors reassure us that this technique captures the pattern they see in particular participants. How does their cross-correlation handle the fact that these are polar histograms (i.e., how do they avoid edge effects?). Some previous papers use a Von Mises distribution to capture the circular nature of these.

We have revised the text to make it clearer that t-tests are done on the results of the circular cross-correlation method (termed “direction distribution displacements", see section 5.1 lines 255-256 and section 5.2 lines 324-325). Additionally, we have included text to the Methods that more carefully describes the circular cross-correlation procedure (section 4.5 lines 188-207). In brief, edge effects are avoided by implementing a normal cross correlation on data that are replicated three times so as to cover the range from -360{degree sign} to 720{degree sign} instead of only 0{degree sign} to 360{degree sign}. Then we search over a finite range for a maximum correlation to avoid spurious peaks given the 180{degree sign} or 90{degree sign} symmetry of the distributions.

We have added a figure to the manuscript showing the polar distribution and resulting cross correlation for a single subject as an example of the method (new Figure 4). We have also added new panels to several figures in the manuscript showing the individual displacements including error bars. See also answer to the comment below regarding bootstrapping.

When we attempted to apply Von Mises fits to our data, we found that the fits on individual subjects were not always reliable (and were complicated by the fact that some individuals showed a vertical saccade bias in addition to a horizontal saccade bias - i.e., some subjects need two lobes while others need four). Thus, we decided to use the cross-correlation approach instead.

2a. One suggestion: Instead of binning saccade directions into bins of 10deg and then interpolating, it might be more precise to apply a kernel density estimate to the saccade directions (of course, this requires a KDE on circular data). Especially the head-orientation vs retina-orientation analysis is on a range where the bins are quite large compared to the effect size. But this point is only a suggestion, not a request.

We thank the reviewer for this suggestion. We have implemented this approach on our data and have found that the results following the cross-correlation analyses using the KDE method are comparable to the results found using the previous histogram and interpolation method. We have implemented this new KDE approach throughout the paper and have revised all the figures and results. We have also added information to the Methods section about this approach (section 4.5 lines 188-207).

2b. At the heart of all analyses in this paper is the distribution displacement, which is computed by maximizing the cross correlation between one distribution and another shifted distribution. Since this value is quite important, I think it should be be inspected a bit more to justify its informativeness. Two things come to mind first:

2a1. checking that the maximum correlation is actually high and that it’s higher than for other shifts. The plotted histograms suggest that that’s the case (which is why I’m not too worried about it), but maybe it should be discussed in the text that this could be a problem, but that it is (likely) not the case here.

We thank the reviewers for the chance to clarify and justify our use of the circular cross correlation method used to obtain direction distribution displacements. The below figure should help to clarify that the maximum correlation between head upright and head right for fractal viewing is indeed high and that it is higher than for other shifts (lags). In the figure, each subject has three panels: the first shows the saccade direction distribution (via KDE) for head tilt right (blue) and head upright (orange) during fractal viewing, the second panel shows the unwrapped distributions from 0{degree sign} to 360{degree sign}, and the third panel shows the circular cross correlation where a maximum is identified. If it had been the case that subjects had circular distributions, then finding a lag at which the correlation is highest would have been difficult, as the reviewer suggests. Some subjects do have less anisotropic distributions than others and thus have less precise measurements of distribution displacements than others. We have added text to the Methods section on these points (section 4.5 lines 198-200).

2a2. Secondly, right now there is no error computed for the displacement, but in theory, there could be quite some error (especially if the distributions are close to uniform -- which they are not). The most natural way to compute an error range for the displacement that comes to my mind (but maybe the authors have an even better idea!) is as follows: We compute the binned histograms for both datasets as done before (let’s say for upright and +30deg). But now, before computing the displacement, we sample from both histograms new datapoints: exactly as many samples as the original data had, which went into said histogram. Then we can compute new histograms from the sampled data and again get two histograms, one for upright and one for +30deg. And now we compute the displacement value for these histograms from the sampled data. We can repeat the procedure of sampling, binning and computing of the displacement value many times and that way bootstrap an error interval for the displacement value. This error will tell us, how much we can rely on the displacement value given that we used only finite data to estimate the underlying distributions. Optimally, these error values would even propagated into the t-test, but that would require a different test and to be honest I’m not sure what the correct test would be, it might require doing Bayesian inference on a graphical model. So I guess it’s fine to keep the t-test as long as there is an discussion why we can trust the displacement values, for example via the mentioned computation of error ranges.

We thank the reviewer for this thoughtful comment. We agree that obtaining error bars for individual subjects provides a more nuanced view into the cross-correlation displacement values. We have implemented the bootstrapping method as suggested on individual subjects for each analysis section in the manuscript by sampling with replacement and calculating displacements a thousand times. We have included a new panel in each of the main figures (Figure 6, 8, and 9) to show individual subjects’ 95% CI bootstrapped error bars. As an example, below is the revised Figure 9, which is the figure that describes the effect of natural scene tilt on saccade directions while the head is simply upright.

Additionally, we have followed the recommendation to add information to the Methods as to the trustworthiness of the direction distribution displacements, evidenced by the bootstrapped error bars, and kept the original statistical strategy of the manuscript for the main effects.

3. When the t-test comparisons are performed, it wasn’t clear why the comparison was upright and right vs. upright and left. Perhaps the effect of interest is just whether the displacement compared to upright is significantly different to zero, not whether this is different in left or right tilts.

Yes, we agree with the reviewer that the main effect of interest is whether the combined displacement from both tilts compared to upright is significantly different from zero. We have now clarified that we calculated the Reference Frame Index (RFIndex) by collapsing the right and left tilts onto each other to obtain a single number that characterizes the overall effect (section 4.5 lines 208-219). We used this index in t-tests for each analysis section.

4. In section 5.3, the RF index result is “somewhat ambiguous” in that it is half way between the image and head hypotheses. This is supported by two t-tests comparing it to 0 and 1 (both significant). In the previous section, only one of these tests is presented and the conclusion is more definitive - that’s a bit inconsistent. In the discussion, the authors suggest that their results are different from previous studies that showed a strong scene-centred bias. That is difficult to judge - have previous studies used the same statistical methods or RF index? What did they find? Is it the case that saccades don’t rotate as much as in previous studies, or that some people do and some people do not? I think it would be beneficial to plot all the conditions for the scene condition (I think that is a 3 x 3 design) so that readers can judge the effects of the two tilts.

We thank the reviewer for this comment. We have added the additional t-test in section 5.2 and rephrased our conclusion of the section. Now we state (lines 424-427): “This result indicates that when individuals view an upright natural scene while their head is tilted, saccades do not precisely follow the orientation of the image or the orientation of the head. Instead, saccade directions fall somewhere in the middle, which suggests that head orientation matters even when viewing an Earth upright scene.”

We have updated the discussion to clarify that the observed discrepancy is based on qualitative analysis of polar distributions reported in previous studies. The difference between our data on saccade directions as a function of scene tilt and previous data on saccade directions as a function of scene tilt is the extent of rotation of the saccade direction distributions. For example, Figure 4 in Foulsham et al., 2008 (also Figure 5 in Bischof et al., 2020) shows all saccades (across all subjects) and the resulting distributions, using 36 bins of 10 degrees each. Those distributions look very precisely aligned with the scene, while our distributions are aligned with the scene, but less so. We noticed in our data that some individual subjects rotate close to the rotation of the images while others do not rotate as much, and we attribute most of this attenuation of the rotation on a group level (compared to other studies) to this fact. The new panels showing the individual direction distribution displacements with the bootstrapped error bars highlight this point in the updated manuscript.

Lastly, below are two figures: the first is a 3 x 3 figure of scene tilt and head tilt as requested, and the second is a figure showing the effect of image tilt across head tilts. We now report in the manuscript that the effect of image tilt relative to the head is comparable across head tilts, per a one-way ANOVA analysis (F(2,39) = 2.96, p = 0.06).

5. Was there a reason this study was performed in VR? Would the same results be expected if images were just presented on a flat screen?

Conducting the study in VR allowed us to track eye movements in eccentric head positions. It also allowed us to totally immerse subjects in the scene, thereby removing the influence of the natural (upright) visual external environment. We would not expect that the results would have changed drastically had we managed to create a setup with flat screen presenting the stimuli and absent external visual cues.

6. The significance statement seems more like a short summary to me. Not sure about expectation due to journal, but I would hope for something that reports not only the results, but why they are important/why I should read the paper. Only last sentence of the significance statement touches on this. But I might simply be misunderstanding what the significance statement is about.

We have consulted the current issue of the journal for examples of significance statements in efforts to revise ours. It seems that most statements in the current issue generally summarize the study in plain language. The eNeuro website states: “The Significance Statement should provide a clear explanation of the importance and relevance of the research in a manner accessible to researchers without specialist knowledge in the field and informed lay readers.” We have added an additional sentence to the end of our statement, which reads: “Future work should consider the influence of head orientation when predicting saccade landing points or when using existing saccade generation models.”

7. I am not sure I correctly understood what Delta_+-30 is in the computation of the RFindex. From the previous section, I guess that Delta_alpha is the Delta between head-upright and head-tilt of alpha, i.e., the displacement in degree that you need to apply to the head-upright data to yield maximum crosscorrelation with the data from the head tilt of alpha? It might be helpful to introduce not only Delta, but also the subscripted version.

We apologize for the unclear notation. We have edited the Methods section (4.5 Data Analysis) to clarify that is meant to represent the direction distribution displacement. More specifically, it represents the direction distribution displacement between 30{degree sign} and 0{degree sign} tilt (30) and between -30{degree sign} and 0{degree sign} tilt (-30). Following this section and equation, the term “direction distribution displacement” is clearly stated in the rest of the manuscript.

8. Right now, the results and the discussion mainly focuses on whether or not the tested effects are significant. I personally think that such a discussion should always be combined with a discussion of how large the effect is. For example, the authors correctly point out that they found an effect of head orientation on saccadic directions even in the scene viewing case (line 281 and line 328). But, unlike in the fractal viewing case, here the effect seems to be very small compared to the effect of the scene directions. This doesn’t make the results wrong or less interesting but I think it helps putting them into a bigger picture.

We thank the reviewer for this suggestion and have incorporated Cohen’s D effect sizes into each of the Results sections of the manuscript.

9. line 396, “However, allocentric maps must also be created and used in saccade generation since we know that saccade directions are influenced by the orientation of scenes and allocentric and egocentric maps are combined in the frontal eye field”. It seems the influence of scene orientation on saccadic directions could in theory also come purely from the alignment of interesting (“salient”) object in the scene. For example, if most interesting stuff is aranged roughly in a line, then most saccades have to be in the orientation of the line, independent on whether the saccades are created in allocentric or egocentric maps.

This is a good point -- it is true that the influence of scene orientation on saccadic directions could originate from simply the alignment of interesting objects in a scene. We have added this possibility to our discussion together with other possibilities such as scene statistics or the perception of gravitational upright. We have added text to the Discussion on this point (section 6, lines 595-598).
